# Mindfulness, Age and Gender as Protective Factors Against Psychological Distress During COVID-19 Pandemic

**DOI:** 10.3389/fpsyg.2020.01900

**Published:** 2020-09-11

**Authors:** Ciro Conversano, Mariagrazia Di Giuseppe, Mario Miccoli, Rebecca Ciacchini, Angelo Gemignani, Graziella Orrù

**Affiliations:** ^1^Department of Surgical, Medical and Molecular Pathology, Critical and Care Medicine, University of Pisa, Pisa, Italy; ^2^Department of Clinical and Experimental Medicine, University of Pisa, Pisa, Italy

**Keywords:** mindfulness, COVID-19, pandemic, meditation, psychological distress, SCL-90, MAAS, adjustment

## Abstract

**Objective:**

Mindfulness disposition is associated with various psychological factors and prevents emotional distress in chronic diseases. In the present study, we analyzed the key role of mindfulness dispositions in protecting the individual against psychological distress consequent to COVID-19 social distancing and quarantining.

**Methods:**

An online survey was launched on March 13, 2020, with 6,412 responses by April 6, 2020. Socio-demographic information, exposure to the pandemic, and quarantining were assessed together with psychological distress and mindfulness disposition. Multivariate linear regression analysis was performed to study the influence of predictive factors on psychological distress and quality of life in Italian responders during the early days of lockdown. Pearson correlations were calculated to study the relationship between mindfulness and psychiatric symptoms.

**Results:**

Multivariate linear regression run on socio-demographics, COVID-19-related variables, and mindfulness disposition as moderators of overall psychological distress showed that mindfulness was the best predictor of psychological distress (β = −0.504; *p* < 0.0001). High negative correlations were found between mindfulness disposition and the overall Global Severity Index (*r* = −0.637; *p* < 0.0001), while moderate to high associations were found between mindfulness and all SCL-90 sub-scales.

**Discussion:**

Findings showed that high dispositional mindfulness enhances well-being and helps in dealing with stressful situations such as the COVID-19 pandemic. Mindfulness-based mental training could represent an effective intervention to stem post-traumatic psychopathological beginnings and prevent the onset of chronic mental disorders.

## Introduction

The current global COVID-19 pandemic has negatively impacted mental health worldwide. In order to respond most effectively to this emergency, an immediate international response from mental health professionals is needed (Aafjes-van Doorn et al., 2020; Conversano et al., 2020b; Fisher et al., 2020; Holmes et al., 2020; Muratori and Ciacchini, 2020). Researchers across the world are promptly trying to address this issue by screening the psychological impact of social distancing and quarantining (Brooks et al., 2020; Orrù et al., 2020; Poli et al., 2020). Results from early studies using social media like Twitter and Weibo data have found that posts related to negative emotions and sensitivity to social risks have greatly increased during lockdown (Kwon et al., 2020; Li et al., 2020). Consistent with these findings, another study on the psychological impact of COVID-19 among Italians during the first week of lockdown has found that 40% of participants reported high psychological distress and about 30% showed clinically significant post-traumatic symptoms (Marazziti et al., 2020). Conversely, adaptive defensive functioning has been found associated with better adjustment and fewer post-traumatic symptoms (Di Giuseppe et al., 2020a). In the present study, we have analyzed the impact of mindfulness dispositions as a protective factor against psychological distress.

In reference to the so-called protective factors in a lowered stress impact perspective, the literature identifies some human abilities already described in ancient times (i.e., Buddhism and other contemplative traditions), which may represent some valid tools in dealing with stress. These abilities may vary from different cognitive processes such as attention, memory, and thought. One of these cognitive processes may be represented by *mindfulness*, the experience of awareness which is activated by purposely paying attention to what occurs in the present, with a non-judgmental attitude (Kabat-Zinn, 2015). In other words, mindfulness may be defined as a process involving attention, awareness, and an open-minded acceptance of the present moment; it concerns the quality of consciousness itself and it is not identified with reflective thought but “offers a bare display of what is taking place” in the moment of observation (Shear and Jevning, 1999, p. 204).

Many researchers, in the last 30 years, have studied this human ability, identifying its intrinsic nature in the human being and its possible enhancement through environmental and behavioral training (Brown and Ryan, 2003; Kabat-Zinn, 2003). To date, this mindfulness ability seems to be related to other psychological constructs such as emotional intelligence, vivid perception, receptive attention, personality traits, and defense mechanisms (Salovey et al., 1995; Costa and McCrae, 2008; Marazziti et al., 2015; Di Giuseppe et al., 2019a). Mindfulness has also been proven to represent a good predictor of depression, anxiety, stress, and well-being in association with self-compassion, self-efficacy, and gender (Soysa and Wilcomb, 2015; Conversano et al., 2020a). Dispositional mindfulness has the potential to be used as treatment for stress-related and other mental health disorders (Baer, 2003; Chang et al., 2004) and is effective in the enhancement of immune function (Davidson et al., 2003) and self-regulation, through the training of attention control, emotion regulation, and self-awareness (Tang et al., 2015; Geng et al., 2019). Interestingly, similar findings were shown in recent studies concerning the key role of defense mechanisms and related psychological functions in chronic illness (Di Giuseppe et al., 2018, 2019b, 2020b; Catalano et al., 2019; Conversano, 2019; Merlo, 2019; Marchi et al., 2019; Lenzo et al., 2020; Martino et al., 2019a, b, 2020a). In particular, mood disorders seem to negatively affect physical and psychological response to treatment in chronic patients (Marazziti et al., 2008; Mula et al., 2008; Dell’Osso et al., 2012; Piccinni et al., 2012; Veltri et al., 2012; Martino et al., 2019c, 2020b). Recent studies demonstrated that mindfulness disposition is related to anxiety and depression and this is observable among different clinical populations (Idusohan-Moizer et al., 2015; Zhang et al., 2015; Lam et al., 2020). Several systematic reviews and meta-analyses showed significant beneficial effects on depressive and anxiety symptoms in patients treated with mindfulness-based interventions (Strauss et al., 2014; Chu et al., 2018).

As regards the measurement of mindfulness levels, there are different validated tools available for scientific research, each of which focuses on different operational definitions (Baer et al., 2004; Baer et al., 2006). The Mindful Attention Awareness Scale (MAAS; Brown and Ryan, 2003) is a psychological instrument to assess the presence or absence of attention to and awareness of what is occurring in the present moment for the participants. It focuses on the above-mentioned core characteristic of mindfulness, which has a dispositional quality. From the validation study of the MAAS, this unique quality of consciousness seems to be related to, and predictive of, a variety of self-regulation and well-being constructs such as optimism, satisfaction, vitality, and self-esteem, and negatively correlated with anxiety, depression, impulsiveness, and self -monitoring (Ryan and Deci, 2000; Brown and Ryan, 2003).

In the present study, regarding the Italian population during the COVID-19 pandemic, we sought to (1) identify individuals at higher risk for psychological distress while measuring the weight of mindfulness disposition in protecting their mental health and (2) assess the relationship between mindfulness and several psychiatric symptoms of distress. Regarding the first hypothesis, we expected that socio-demographic characteristics and lockdown duration would negatively affect psychological well-being, while higher mindfulness would lower the levels of distress. Regarding the second hypothesis, we expected that higher mindfulness disposition would be associated with lower self-rated psychiatric symptoms and lower overall psychological distress.

## Materials and Methods

### Participants

From March 13 to April 6, 2020, we collected 6,412 responses from people living in Italy to an online survey about the psychological impact of COVID-19 during the lockdown. Responders were mostly middle-aged adults between 30 and 50 years old, while approximately 33 and 27% were younger and older, respectively. Most of the sample was represented by females, living with close relatives, without children (see Table 1 for descriptive statistics). Participants prevalently came from Central Italy (*N* = 3,463; 54%), whereas 25% (*N* = 1,603) and 21% (*N* = 1,346) were in the North and the South of Italy, respectively. The rates of reported positive cases and deaths among close relatives or friends were about 7% (*N* = 417) and 3% (*N* = 167), respectively.

**TABLE 1:** Descriptive statistics of responders’ socio-demographic characteristics (*N* = 6,412).

		*N*	%
**Age**			
	<30	2,099	32.7
	30–50	2,572	40.1
	>50	1,741	27.2
**Gender**			
	Male	1,604	25.0
	Female	4,808	75.0
**Location**			
	North Italy	1,603	25%
	Central Italy	3,463	54%
	South Italy	1,346	21%
**Living with**			
	Close relatives	4,508	70.3
	Partners	838	13.1
	Roommate	286	4.4
	Alone	780	12.2
**Having children**			
	Yes	2,619	40.8
	No	3,793	59.2
**Positive cases among relatives and friends**			
	Yes	417	7%
	No	5,995	93%
**Deaths among relatives and friends**			
	Yes	167	3%
	No	6,245	97%

### Measures

To evaluate psychological distress and individual adaptive responses we conducted a survey exploring socio-demographic information, COVID-19-related information (i.e., presence/absence of positive cases or death among relatives or friends), psychological distress, and mindfulness disposition. The Italian version of the Symptoms Checklist-90 (SCL-90; Derogatis et al., 1973; Cassano et al., 1999) and the Mindfulness Attention Awareness Scale (MAAS; Brown and Ryan, 2003; Veneziani and Voci, 2015) were used for the assessment of psychological variables.

The *Symptoms Checklist-90* is a 90-item 5-point scale assessing psychopathological and somatic symptoms occurring during the past week. Even if it does not control for deceptive responses (Sartori et al., 2017), in this specific setting is adequate given the absence of faking proneness in respondents. The SCL-90 provides a Global Severity Index (GSI) and nine subscale scores for psychiatric symptoms, such as Somatization (SOM), Obsessive Compulsive Disorder (O-C), Interpersonal Sensitivity (I-S), Depression (DEP), Anxiety (ANX), Hostility (HOS), Phobic Anxiety (PHOB), Paranoid Ideation (PAR), and Psychoticism (PSY). Validity and reliability of the scale have been largely documented (Derogatis et al., 1976; Derogatis and Cleary, 1977; Bonicatto et al., 1997; Lara et al., 2005).

The *Mindfulness Attention Awareness Scale* is a 15-item single-dimension measure of the frequency of open and receptive attention to, and awareness of, ongoing events and experience using a 6-point Likert. All items are presented as negative descriptions of mindfulness, so higher scores indicate less mindfulness. For the purpose of the study, we adapted the MAAS, resulting in a reversed 5-point Likert scale, so higher scores indicated greater mindfulness. The MAAS is a reliable instrument with a Cronbach’s α of 0.87. Adequate test-retest reliability, and convergent as well as discriminate validity have been reported (Black et al., 2012).

### Procedure

An online questionnaire was launched online on March 13, 2020, at 17:00 (GMT + 1), 2 days after the Italian Government Decree of lockdown for slowing the diffusion of the COVID-19 outbreak. Participants were recruited using snowball sampling among all Italian residents living in Italy at the time of data collection. They were informed about the purpose of the study and asked to give their approval on personal data treatment. All procedures followed the ethical standards and were approved by the Ethics Committee of the University of Pisa (n. 0036344/2020).

### Statistical Analyses

Descriptive data are presented as means, standard deviations, 95% confidence intervals, absolute and relative frequencies. The Anderson-Darling test and Normal P-P plot were used to verify normality of distributions. Pearson correlation coefficients were calculated, *t*-test and simple linear regressions were performed to study the relations between the variables and the outcome. Multivariate analysis was carried out to study the influence of predictive factors on psychological distress. Tolerance index and Variance Inflation Factor (VIF) were calculated to verify the level of correlation between predictors. Tolerance values were > 0.5 and VIF values were < 2, these results showed no evidence of multicollinearity. Goodness of fit of the multivariate analysis was verified, adjusted *R*^2^ resulted 0.43, showing a good level of fit model. *Post hoc* power analysis was used to evaluate the sample size and the probability of type II error; the range of statistical power was 0–1 and the power of the sample was 1. The significance level was set to 0.05 and the analyses were performed with R version 4.0.0.

## Results

Table 1 shows descriptive statistics for socio-demographic characteristics of participants. In line with previous studies, young, female, living with parents, and not having children were more frequent among responders. Descriptive statistics for psychological variables are displayed in Table 2. Mindfulness mean scores assessed around normative values for healthy individuals (*M* = 2.881; *SD* = 0.653), while psychological distress mean score ranged slightly below the cut-off for clinical significance (*M* = 0.730; *SD* = 0.536). These results indicated that responders represented a community sample experiencing a stressful life event such as the lockdown as a consequence of the COVID-19 outbreak.

**TABLE 2:** Descriptive statistics of responders’ psychological characteristics (*N* = 6,412).

		Mean	*SD*	95% interval confidence	
				Lower	Upper
Mindfulness (MAAS)		2.881	0.653	2.865	2.897
Psychological distress (SCL-90 GSI)		0.730	0.536	0.717	0.743
	SCL-90 SOM	0.622	0.605	0.607	0.637
	SCL-90 O-C	0.872	0.683	0.855	0.889
	SCL-90 INT	0.607	0.588	0.592	0.621
	SCL-90 DEP	0.977	0.746	0.958	0.995
	SCL-90 ANX	0.860	0.694	0.843	0.877
	SCL-90 HOS	0.650	0.613	0.635	0.665
	SCL-90 PHOB	0.478	0.548	0.465	0.492
	SCL-90 PAR	0.713	0.665	0.697	0.730
	SCL-90 PSY	0.509	0.522	0.497	0.522
	SCL-90 SLEEP	1.076	0.982	1.052	1.100

Tables 3, 4 show results from univariate and multivariate linear regression run on socio-demographic variables and mindfulness disposition as predictors of overall psychological distress are displayed. Table 3 shows results of preliminary univariate linear regression analyses of all socio-demographic variables on the GSI. We entered in the multivariate linear regression model only variables that resulted significantly related to the GSI. Variables included were age, gender, quarantine, people living with, having children, and mindfulness. Table 4 shows that all factors resulted significantly, with the only exception of having children that was no longer significant in the final model. Mindfulness resulted the best predictor of GSI, about 10 times more effective than other predictors included. Each increase of one unit of MAAS results in a decrease of -0.5 in GSI (β = -0.504; *p* < 0.0001). Conversely, quarantining negatively affected mental health, increasing GSI of 0.05 for each week passed in lockdown. Younger subjects and females were at higher risk for mental health problems, while living with one’s spouse resulted in them feeling slightly protected in terms of psychological well-being. VIF < 2 demonstrated low collinearity level between independent variables.

**TABLE 3:** Univariate linear regressions for socio-demographic variables and mindfulness predicting psychological distress.

	Global Severity Index (GSI)					
	β	*SE*	*t*	*P*	95% CI	
Age	–0.072	0.004	–16.120	<0.0001	–0.081	–0.063
Gender (female)	0.175	0.015	11.436	<0.0001	0.145	0.205
Having children	–0.141	0.013	–10.446	<0.0001	–0.167	–0.114
Location Central Italy	–0.017	0.018	–0.996	0.319	–0.052	0.017
Location South Italy	–0.039	0.021	–1.863	0.063	–0.079	0.002
Positive cases	0.046	0.027	1.682	0.093	–0.008	0.099
Deaths	0.040	0.042	0.939	0.348	–0.043	0.122
Living with lover	0.133	0.033	4.079	<0.0001	0.069	0.197
Weeks in lockdown	0.052	0.008	6.183	<0.0001	0.036	0.069
Mindfulness (MAAS)	–0.504	0.008	–64.169	<0.0001	–0.519	–0.488

*Location Central Italy and Location South Italy resulted non-significant in comparison with Location North Italy.*

**TABLE 4:** Multivariate linear regression for socio-demographic variables and mindfulness predicting psychological distress.

	Global Severity Index (GSI)					
	β	*SE*	*t*	*P*	95% CI	
Age	–0.039	0.005	–8.320	<0.0001	–0.048	–0.030
Gender (female)	0.078	0.012	6.570	<0.0001	0.054	0.101
Living with lover	–0.040	0.016	–2.527	0.012	–0.072	–0.009
Weeks in lockdown	0.050	0.006	7.846	<0.0001	0.038	0.063
Mindfulness (MAAS)	–0.504	0.008	–64.169	<0.0001	–0.519	–0.488
Having children	–0.021	0.014	–1.478	0.140	–0.050	0.007

*Living with lover is significant in comparison to living with parents, whereas either living alone or with roommates resulted non-significant in comparison to living with parents.*

Table 5 shows Pearson correlations between MAAS and SCL-90. High negative correlations were found for mindfulness disposition and overall psychological distress (*r* = -0.637; *p* < 0.0001), obsessive compulsive (O-C; *r* = -0.627; *p* < 0.0001), and psychoticism (PSY; *r* = -0.593; *p* < 0.0001). The remaining SCL-90 sub-scales showed moderate negative correlations ranging from 0.386 to 0.565 (all *p* < 0.0001). In descending order of magnitude they were: depression (DEP), interpersonal sensibility (INT), anxiety (ANX), paranoid (PAR), hostility (HOS), somatization (SOM), phobic anxiety (PHOB), and sleep disturbance (SLEEP), with a very tight 95% confidence interval demonstrating excellent goodness of fit.

**TABLE 5:** Pearson correlations between mindfulness disposition (MAAS) and psychological distress (SCL-90).

	MAAS (*N* = 6,412)	
	*r*	*p*
GSI	−0.637	<0.0001
SCL-90 SOM	−0.466	<0.0001
SCL-90 O-C	−0.627	<0.0001
SCL-90 INT	−0.551	<0.0001
SCL-90 DEP	−0.565	<0.0001
SCL-90 ANX	−0.536	<0.0001
SCL-90 HOS	−0.487	<0.0001
SCL-90 PHOB	−0.415	<0.0001
SCL-90 PAR	−0.533	<0.0001
SCL-90 PSY	−0.593	<0.0001
SCL-90 SLEEP	−0.386	<0.0001

## Discussion

The current study contributes to a growing literature on the benefits of protective factors on psychological functioning during high stress situations. According to our findings, dispositional mindfulness may represent a stable protective factor from the current intensity of mental distress of individuals; in fact, increasing levels of mindfulness corresponded to significantly decreased mental discomfort.

With regard to our first hypothesis which stated that certain individuals are at higher risk for psychological distress, whereas others show better adjustment to stressful situations, the results indicated that dispositional mindfulness, older age, living with loved one, and being a parent were protective factors, while female gender and lockdown duration increased the risk of psychological distress. Each increase of one unit of MAAS results in a decrease of -0.5 in GSI (β = -0.504; *p* < 0.0001), indicating that mindfulness disposition is extremely important as a psychological resource that helps the individual to manage stressful situations (Bränström et al., 2011; Rasmussen and Pidgeon, 2011; Bao et al., 2015; Dixon and Overall, 2016). Conversely, quarantining negatively affected mental health, increasing GSI of 0.05 for each week passed in lockdown. Consistent with previous studies (Di Giuseppe et al., 2019c, 2020c; Carmassi et al., 2014, 2018), younger subjects and females were at higher risk for mental health problems, while living with one’s spouse resulted in them feeling slightly protected in terms of psychological well-being. The protective factor of mindfulness disposition and training has already been shown in previous literature, influencing positively memory function, cognitive resilience, mental health, emotional balance, and high stress functioning, also decreasing anxiety, depression, and burnout levels (Jha et al., 2010, 2017; Abenavoli et al., 2013; De Frias and Whyne, 2015; Westphal et al., 2015; Xu et al., 2017). This particular result is extremely significant as it shows that “being mindful” helps in dealing with stressful situations such as the one we are experiencing at the moment and leaves open the possibility for mental health professionals to use mindfulness-based mental training to stem the post-traumatic psychopathological beginnings that are likely to manifest in the future.

In addition to mindfulness, other protective factors emerged from our investigation. Findings showed that with increasing age, individuals showed less psychological distress. As other studies have already demonstrated, age of onset is exceedingly early for some psychopathological disorders such as anxiety, substance use, impulse control, and mood disorders (Kessler et al., 2007; Jones, 2013). Nevertheless, it has been proven that after an emergency due to natural disasters, older adults have increased resiliency to psychopathologies such as post-traumatic stress disorder, mood, and anxiety disorders (Acierno et al., 2006). Taken together, our results confirm a greater resilience in adults compared to young people with regard to high stress functioning, providing an important reflection on the direction of the efforts that mental health professionals will have to address their attention to soon. Furthermore, the social condition (cohabitants’ situation during quarantine) could represent a protective factor against psychological distress. Not surprisingly, amongst the four conditions proposed in our survey, “living with a partner” predicted lower distress during the COVID-19 quarantine. According to previous studies, social relations are indeed a protective factor for mental health, increasing resilience (Fuller-Iglesias et al., 2008) but our findings showed that to be significant, it should come from loved ones. In fact, separation from loved ones seems to be the crucial psychological distress factor during quarantine, despite the support of new devices that are keeping us connected (Maunder et al., 2003; Manuell and Cukor, 2011; Brooks et al., 2020). Furthermore, ‘being a parent’ seems to be a protective condition against mental illness, as already reported in other studies (Helbig et al., 2006; Nelson et al., 2014).

Conversely, gender and lockdown duration were good predictors of higher distress among Italians. Female gender seems to be solidly related with higher psychological distress than male gender. As well known, men and women experience different kinds of mental health problems; females tend to exceed males in internalizing disorders with higher prevalence of depression and anxiety (Rosenfield, 1999; Rosenfield and Mouzon, 2013). This gap narrows with the temporal and spatial variation in gender role traditionality (Seedat et al., 2009). There is no doubt that particular attention should be paid to women in this crucial period, who, in addition to being at greater risk of developing a mood disorder or suffering from psychological distress, are unfortunately the main victims of domestic violence (Bradbury-Jones and Isham, 2020; Taub, 2020). Furthermore, the economic downturn caused by this emergency is more likely to impact sectors with high female employment shares, together with the increasing needs in childcare (Alon et al., 2020). Finally, our findings suggested that lockdown duration has a negative impact on mental health; a previous study displayed in the first phase of the Italian quarantine had already reported that individuals in quarantine experienced negative psychosocial changes such as sleep disturbances, post-traumatic symptoms, depression, and anxiety which all have a massive impact on well-being (Brooks et al., 2020; Cellini et al., 2020; Wang et al., 2020; Di Giuseppe et al., 2020d).

Our second hypothesis of a strong negative relationship between mindfulness and psychiatric symptoms of distress was fully confirmed. Mindfulness was found to be negatively correlated with all the analyzed subscales. In particular, individuals with lower mindfulness disposition are more likely to present thoughts, impulses, and actions that are experienced as irresistible but are of an unwanted nature, and cognitive attenuation (Obsessive-compulsive subscale). In addition, lower mindfulness predicted the experience of withdrawal, isolation, and a schizoid lifestyle and also on first-rank schizophrenia symptoms such as hallucinations and thought-broadcasting (Psychoticism subscale; Matti, 2003). Moreover, significant moderate correlations were found for all psychiatric symptoms assessed with the SCL-90, with greater magnitude on interpersonal sensitivity, depression, and anxiety. Results demonstrated that a higher disposition toward mindfulness may represent a protective factor against anxiety, dysphoric mood, lack of motivation, loss of vital energy, feelings of hopelessness, and cognitive and other somatic correlates of depression, as already shown in previous studies (Teasdale et al., 2000; Evans et al., 2008; Walsh et al., 2009; Deng et al., 2014; Segal and Teasdale, 2018).

The present study has several strengths and innovative features, as well as a number of limitations. First, the cross-sectional research design does not allow us to determine causal relationships between studied variables (Pearl, 2010; Orrù et al., 2020, 2020a, b). Moreover, we used a non-randomized sampling method, the so-called snowball sampling, which could be possibly biased by uncontrolled variables. Furthermore, all measures are self-reported and thus they might be biased by the responders’ self-observation attitude. Finally, psychological information was collected online and without any independent evaluation of the health status of the respondents.

Despite all the above, this study indicates that mindfulness and socio-demographic characteristics play a key role in moderating the experience of COVID-19-related distress. Mindfulness dispositions and practice, as with other psychological resources (Lingiardi et al., 2010; Fonagy and Bateman, 2016; Prout et al., 2019; Di Giuseppe et al., 2020e), enhances adaptation and leads to a better adjustment to stress (Maffei et al., 1995; Di Giuseppe et al., 2019a; Marazziti et al., 2020). In order to stem the psychopathological consequences lying ahead in the future due to the traumatic nature of the recent pandemic and quarantine, we therefore suggest paying accurate attention to mindfulness disposition and training, with the purpose of enhancing resilience to high-stress events and preventing the onset of chronic mental disorders.

## Data Availability Statement

The raw data supporting the conclusions of this article will be made available by the authors, without undue reservation, to any qualified researcher.

## Ethics Statement

The studies involving human participants were reviewed and approved by the Ethics Committee of the University of Pisa. The patients/participants provided their written informed consent to participate in this study.

## Author Contributions

CC and MD conceived the assessment. MM contributed to the data analysis. RC contributed to the data interpretation. CC, MD, MM, RC, AG, and GO drafted the manuscript. All authors critically revised the manuscript and approved the final version to be published.

## Conflict of Interest

The authors declare that the research was conducted in the absence of any commercial or financial relationships that could be construed as a potential conflict of interest.
